# Factors associated with health-seeking behavior among migrant workers in Beijing, China

**DOI:** 10.1186/1472-6963-10-69

**Published:** 2010-03-19

**Authors:** Yingchun Peng, Wenhu Chang, Haiqing Zhou, Hongpu Hu, Wannian Liang

**Affiliations:** 1 School of Health Administration and Education, Capital Medical University, Beijing 100069, China; 2 School of Public Health, Peking University, Beijing 100083, China; 3 Office of Health Emergency, Ministry of Health, Beijing 100044, China

## Abstract

**Background:**

Migrant workers are a unique phenomenon in the process of China's economic transformation. The household registration system classifies them as temporary residents in cities, putting them in a vulnerable state with an unfair share of urban infrastructure and social public welfare. The amount of pressure inflicted by migrant workers in Beijing, as one of the major migration destinations, is currently at a threshold. This study was designed to assess the factors associated with health-seeking behavior and to explore feasible solutions to the obstacles migrant workers in China faced with when accessing health-care.

**Methods:**

A sample of 2,478 migrant workers in Beijing was chosen by the multi-stage stratified cluster sampling method. A structured questionnaire survey was conducted via face-to-face interviews between investigators and subjects. The multilevel methodology (MLM) was used to demonstrate the independent effects of the explanatory variables on health seeking behavior in migrant workers.

**Results:**

The medical visitation rate of migrant workers within the past two weeks was 4.8%, which only accounted for 36.4% of those who were ill. Nearly one-third of the migrant workers chose self-medication (33.3%) or no measures (30.3%) while ill within the past two weeks. 19.7% of the sick migrants who should have been hospitalized failed to receive medical treatment within the past year. According to self-reported reasons, the high cost of health service was a significant obstacle to health-care access for 40.5% of the migrant workers who became sick. However, 94.0% of the migrant workers didn't have any insurance coverage in Beijing. The multilevel model analysis indicates that health-seeking behavior among migrants is significantly associated with their insurance coverage. Meanwhile, such factors as household monthly income per capita and working hours per day also affect the medical visitation rate of the migrant workers in Beijing.

**Conclusion:**

This study assesses the influence of socio-demographic characteristics on the migrant workers' decision to seek health care services when they fall ill, and it also indicates that the current health service system discourages migrant workers from seeking appropriate care of good quality. Relevant policies of public medical insurance and assistance program should be vigorously implemented for providing affordable health care services to the migrants. Feasible measures need to be taken to reduce the health risks associated with current hygiene practices and equity should be assured in access to health care services among migrant workers.

## Background

The inception of China's reform and opening policy three decades ago has resulted in the creation of a growing, historically unique social group: rural migrants in the big cities. According to statistical data, the rural migrant population in China rose from 70 million in 1993 to 140 million in 2003, doubling the original number in 10 years and including nearly 30% of the rural labor force [1]. Rural migrants have grown into an important new social stratum of China, particularly as a sizeable component of industrial workers in China and a driving force for urbanization. Investigations indicate that over 60% of rural migrant workers swarm into large cities so that many Chinese metropolises, such as Beijing, Shanghai and Guangzhou become overloaded. Beijing, the capital city of China, is a major migration destination, where the migrant population currently exceeds 4 million, accounting for 1/4 of the total population [2]. As most migrants can only do simple, unskilled jobs, typically unstable and insecure work, they are often low paid and frequently laid off. Gradually they become the growing poor group in the big cities, lacking a social assistance system and public infrastructure and suffering from health risks which go unnoticed [3].

Although rural migrant workers have contributed much to urban and national economic development, a series of existing problems drag them into "vulnerable groups"[4]. China's national policy has long been established on locality-based schemes. According to law all individuals must have household registration *(hukou)*, by which certain rights, such as free education and access to social welfare, are offered. Since household registration is not easily transferable from rural to urban areas, migrants are rarely entitled to public medical insurance and assistance programs in places outside their original residential area, forcing them to pay out-of-pocket expenses for medical services in cities [5,6].

While available studies have primarily focused on AIDS and tuberculosis, or reproductive health of female migrants, only limited research has been conducted on health care access and the health-seeking behaviors of this population [7,8]. Systematic research on how Chinese rural migrants perceive health, disease, and the health care system is far from sufficient. It behooves us to understand how this group perceives the various possibilities for health care: self-medication, private clinics with varied levels of care, and more formal hospital treatment. The concept and awareness of health risks and the knowledge of medicine as well, play a big part in health-related behaviors of the migrants. Understanding these factors will be crucial to prevention, intervention, and other health-related measures for the migrant workers in China [9].

With violently physical, demographic and socioeconomic changes, Beijing is typical of the big cities undergoing market transition and economic restructuring. Therefore, it is an ideal case to study the migrant population against the background of market transition in China. This paper is to collect information about the attitude, perception, preferences and health-seeking behavior among the migrant workers in Beijing. Our present study will also assess their needs in health care services and the obstacles they meet in enjoying health services as well as suggest feasible solutions to presented problems. It is also hoped that policy implications on the management of the issues related to the migrant workers can be drawn from the analysis.

## Methods

### Research sites

About 60 percent of the migrant population lives in three of eight urban districts of Beijing, i.e. Chaoyang, Haidian and Fengtai[10]. Chaoyang disrict has the highest number and density of migrants among all, owing to its rapid commercialization in recent years. Its migrant population of 1 million accounts for 1/4 of all the migrants in Beijing, and nearly a third of all the residents in that district. The three districts listed above were chosen for this study.

### Data Collection and Measures

According to the Beijing Municipal Regulations on migrants, migrants must register with the local community agencies where they are living to receive and renew their temporary residency certificates. The migrants tend to congregate among themselves according to their hometown (*laoxiang guanxi*), and live in so called 'migrant villages' *(liudong renkou jujudian) *dispersed among the city districts. A multi-stage stratified cluster sampling method was used in this study. In the first stage, for having the largest and densest migrant populations among all districts, the three districts Chaoyang, Haidian and Fengtai were selected from Beijing. In the second stage, 5 to 8 towns were chosen from each selected district according to the number of migrants. In the third stage, 1 to 2 'migrant villages' from every selected town were chosen according to geographical origin of the migrants, i.e. where they used to live. Finally, based on records of migrants kept in the community agencies, all eligible individual participants were chosen from the selected 'migrant villages'. The criteria for selecting participants were: 1) They were aged from 15 to 65 years old; 2) They had been living in Beijing for at least 3 months; 3) They weren't registered as permanent residents in Beijing.

With the help of local community agencies in these three districts, the interview was conducted face -to -face between interviewers and interviewees at their temporary places of residence in Beijing during their free time.

The study team set up a strict process of review and supervision to ensure survey quality. All of the interviewees were assured that they would remain anonymous during the interview and the analysis. The right to refuse participation was guaranteed. Informed consent was obtained from each participant at the start of the interview, and all participants received a gift following. This study was approved by the ethics committee of Capital Medical University.

The structured questionnaire survey was conducted by a team of trained investigators from March to April, 2008 in the three districts selected in Beijing. A total of 2,545 migrant workers were enrolled in the study. The questions covered such areas as social demographic features, perceived health status, health insurance coverage, health-seeking behavior, perceptions of health risk and so on.

The questionnaire was developed in three steps: Firstly, the items of the questionnaire were generated from literature research. Secondly, they were evaluated by a formal consensus process based on a nominal group technique (NGT), which is a structured variation of small group discussion methods. The process prevents the domination of discussion by a single researcher, encourages the more passive group members to participate, and results in a set of prioritized solutions or recommendations [11,12]. Lastly, the questionnaire was piloted on 94 migrant workers from Chaoyang district. The internal consistency reliability (Cronbach's α) for the full scale was 0.812. Meanwhile, face validity and content validity of the questionnaire were both confirmed by epidemiologists and experts from health administration. The results indicated that the questionnaire had good reliability and validity.

### Statistical Analysis

Prior to the analysis, all questionnaires were reviewed for accuracy. Two research assistants independently uploaded the data into a computerized database using EpiData3.0. Chi-square test was performed to analyze differences in the health- seeking behavior between different socio-demographic groups of migrant workers. The difference was considered statistically significant if the 2-sided *P *value was less than 0.05. As a hierarchy exists in the dataset, a multilevel methodology (MLM) is used to demonstrate the independent effects of the explanatory variables on health seeking behavior in the final models, the parameter estimates are exponentiated and interpreted as relative risks. Among the multilevels, level 1 is individual, level 2 is 'migrant village', level 3 is town, and level 4 is district, respectively.

All analyses were performed using the MLwiN software package(MlwiN version 2.02, Rasbash J et al,2005) and SPSS for Windows, version 12.0(SPSS Inc., Chicago, IL).

## Results

### Socio-demographic characteristics of the respondents

A total of 2,545 migrant workers were enrolled in the study, excluding incomplete data, 2,478 valid respondents were received (97.4% response rate). 71.4% of respondents lived in Chaoyang District, 13.2% in Haidian District, and 15.4% in Fengtai District.

As Table 1 shows, of the 2,478 participants aged from 15 to 65, their average age was 33.9 (standard deviation (SD) 7.2), 71.2% aged from 20 to 39 years old, and 57.2% are males. 11.3% of them were illiterate or almost illiterate. The wholesale or retail sector and lodging catering service sector were the major employers of employed groups, and accounted for 28.3% and 19.9% of the respondents, respectively.

**Table 1:** Socio-demographic characteristics of migrant workers in Beijing (n = 2478)

Variable	n	%
**Gender**		
Female	1060	42.8
Male	1418	57.2
**Age(years)**		
15-19	88	3.6
20-29	764	30.8
30-39	984	40.4
40-49	468	18.9
50-59	124	5.0
Over 60	152	1.3
**Education**		
Illiteracy	280	11.3
Primary school	528	21.3
Secondary school	1206	48.7
High school	406	16.4
University/college degree	58	2.4
**Occupation**		
Wholesale and retail	702	28.3
Lodging catering service	494	19.9
Construction	454	18.3
Manufacturing	320	12.9
Transport, storage and postal	302	12.2
Domestic service	112	4.5
Others	94	3.8
**Working hours per day (hours)**		
Less than 8	494	19.9
8-9	550	22.2
10-11	672	27.1
12-13	594	24.0
Over 13	168	6.8
**Monthly household income per capita (RMB)**		
Less than 250	98	4.0
251-500	152	6.1
501-750	640	25.8
751-1000	928	37.4
1001-1250	410	16.5
Over 1250	250	10.1
**Duration of stay in Beijing (years)**		
Less than 1	520	21.0
1-5	1549	62.5
Over 5	409	16.5
**Insurance coverage**		
None	2329	94.0
Employer-based medical insurance	57	2.3
Commercial medical insurance	27	1.1
Social health insurance	22	0.9
Industrial injury insurance	2	0.1
Others	40	1.6

*Note: *Table 1 presents the socio-demographic characteristics of all the migrant workers that participated in the study, the total sample is 2,478.

The survey also showed that migrant workers' average working time was 6.2 days per week, 10.3 hours per day. 30.8% of the total subjects worked more than 12 hours per day. It was found that the migrants' monthly household income per capita was 842 Yuan RMB, among them 10.1% made less than 500 Yuan, and only 10.1% made more than 1,250 Yuan.

Of all the migrants investigated, 58.2% lived with their family members in the 'migrant villages'. 96.9% of the subjects lived in rental houses, and 2.5% lived in dormitory-style accommodations provided by their work units, sharing with colleagues, and where there exist public toilet facilities.

This survey showed that 2,329(94.0%) of the total respondents had no any insurance coverage in Beijing, only 2.3% had employer-based medical insurance, 1.1% had commercial medical insurance, 0.9% had social health insurance, and 0.1% had industrial injury insurance.

### Health seeking behavior and risk perceptions among migrant workers

According to self-rated health statuses, 1,135 (45.8%) of the respondents felt very good, while only 2.3% felt bad and 0.2% felt very bad. As to the preferable medical institutions they would consult, 504 (20.3%) of the total subjects replied they never seek any treatment in medical institutions in Beijing. Of the remaining subjects, 31.6% selected village health clinics or community health service stations(Table 2), while 172 (6.9%) selected so-called private clinics (according to the supervised records kept in the local health bureau, most of the so-called clinics in the 'migrant villages' of the three districts were unlicensed, and the service providers were unqualified practitioners in Beijing). Regarding the reasons why they selected so-called private clinics, the relatively lower medical expenses (48.8% of 172 subjects) and easier access to health services (36.0%) were mainly mentioned.

**Table 2:** Self-reported preferred medical institutions by migrant workers when fallen ill in Beijing

Medical institutions	n	%
Not seeking care in medical institutions	504	20.3
Village health clinics or community health service stations	784	31.6
Township hospital	358	14.4
District-level hospital	266	10.7
City-level hospital or above	362	14.6
Unlicensed private clinic	172	6.9
Others	32	1.3
Total	2478	100.0

*Note: *Table 2 presents the self-reported preferred medical institutions by migrant workers when fallen ill in Beijing, 504 of the total subjects replied they would never seek any health care from medical institutions **(n = 2478)**.

Concerning what measures would usually be taken when they fell ill in Beijing (In this study, illness means sickness or impairment that often affects a whole body or whole system), 316 (11.8%) of the total subjects replied they had not so far fallen ill, 66.1% of the remaining 2,162 respondents answered that they would see a doctor, 27.6% would take self-medication, 3.7% would have a rest, and another 2.6% wouldn't take any measures. As to why the migrant workers wouldn't visit a doctor when ill, 66.2% of 732 subjects answered that they felt it's not a big trouble, 8.9% admitted that they were unable to pay medical expenses, 7.0% said that it was due to the unreasonable charges in medical institutions, 5.5% expressed having no free time, and 6.2% thought they knew how to deal with illness themselves (Table 3).

**Table 3:** Self-reported main reasons for not seeking health care among migrant workers when fallen ill in Beijing (n = 732)

Main reasons	n	%
Feeling their own diseases not severe enough	485	66.2
Unable to pay medical expenses	65	8.9
Unreasonable charges in medical institutions	51	7.0
Knowing how to deal with diseases themselves	45	6.2
Having no free time	40	5.5
Long distance from medical institutions	13	1.8
Complicated medical procedures	13	1.8
Long queuing and waiting time	12	1.6
Poor service attitude and discrimination	4	0.6
Excessive service	1	0.1
Others	2	0.3
Total	732	100.0

*Note: *316 of the total subjects replied they had not yet gotten sick, 1,430 (66.1%) of the remaining 2,162 respondents (2,478 minus 316) answered that they would see a doctor, while the remaining 732 subjects (2,162 minus 1,430) would not seeking health care, the reasons were shown in Table 3.

As table 4 shows, 309 (324 incidents) of the total respondents fell ill within the past two weeks (two-week prevalence rate of the migrants was 13.1%). Of 324 incidents, 36.4% had seen a doctor (two-week visitation rate of the total sample was 4.8%), 33.3% had taken self-medication, while 30.3% hadn't taken any measures. Reasons as to why subjects didn't see a doctor were due to inability to afford the high medical expenses (40.5% of 206 subjects), neglect of the severity of the diseases (33.4%) and no free time (26.1%). Among those who ever visited a doctor, 44.6% had chosen to go to village health clinics or community health service stations, while 20.0% selected the so-called private clinics.

**Table 4:** Health seeking behavior within the past two weeks among migrant workers who fell ill and visited doctors

Item	Person-time	%
**Utilization of health services**		
Saw a doctor	118	36.4
Self-medication	108	33.3
Non use	98	30.3
Total	324	100.0
**Type of selected medical institutions**		
Village health clinics or community health service stations	53	44.6
Township hospital	7	6.2
District-level hospital	20	16.9
City-level hospital or above	15	12.3
Unlicensed private clinic	24	20.0
Total	118	100.0

*Note: *309 (324 incidents) of the total respondents fell ill within the past two weeks, while only 4.6% (118) of the respondents had visited a doctor.

Within the past twelve months, 4.6% (114) of respondents had received hospital inpatient care, of which 42 (36.8%) selected hospitals in Beijing and 72 (63.2%) selected hospitals in their hometown. The reasons as to why some didn't select hospitals in Beijing include high medical expenses (52.7% of 72 subjects), inconvenient and inaccessible services (28.6%), and being discriminated against (4.8%). Meanwhile, 28 interviewees of 142 subjects who were recommended by their doctors to be hospitalized were not admitted to a hospital within the past twelve months(from 1 Mar,2007 to 28 Feb,2008), the ratio of without hospitalization to those who should have been hospitalized was 19.7%. Of this group, 14 (50.0%) answered they were unable to afford the high medical expenses and 33.3% thought their illness weren't so severe.

### Analysis of factors associated with health seeking behavior among migrant workers in Beijing

In order to explore the factors associated with health seeking behavior among migrants, Chi-square (χ^2^) tests were firstly conducted to compare the differences between migrant workers with different socio-demographic characteristics. As table 5 shows, no statistically significant difference was found in the health seeking behavior among those of different gender, age, occupation or duration of stay in Beijing. However, differing education level, monthly household income per capita, working hours per day and insurance coverage were found to be statistically significantly associated with the health seeking behavior of migrant workers (*P *< 0.05).

**Table 5:** Association of demographic characteristics and health seeking behavior among migrant workers who fell sick in the past two weeks

Characteristic	Number of fallen sick	Number of those who saw a doctor (%)	***χ***^**2**^	*P *value
**Gender**				
Female	157	58(36.9)	0.036	0.850
Male	167	60(35.9)		
**Age(years)**				
15-19	45	17(37.8)	0.827	0.975
20-29	53	19(35.8)		
30-39	48	21(43.8)		
40-49	57	15(26.3)		
50-59	61	23(37.7)		
Over 60	60	23(38.3)		
**Education**				
Illiteracy	53	12(22.6)	9.857	0.043
Primary school	65	21(32.3)		
Secondary school	145	55(37.9)		
High school	51	24(47.1)		
University/college degree	10	6(60.0)		
**Occupation**				
Wholesale and retail	41	21(51.2)	10.699	0.098
Lodging catering service	52	14(26.9)		
Construction	40	10(25.0)		
Manufacturing	46	15(32.6)		
Transport, storage and postal	42	17(40.5)		
Domestic service	58	26(44.8)		
Others	45	15(33.3)		
**Working hours per day(hours)**				
Less 8	70	34(48.6)	15.290	0.004
8-9	60	27 (45.0)		
10-11	56	23(41.1)		
12-13	65	17(26.2)		
Over 13	73	17(23.3)		
**Monthly household income per capita (RMB)**				
Less 250	65	10(15.4)	37.198	0.000
251-500	59	15(25.4)		
501-750	51	16(31.4)		
751-1000	63	26(41.3)		
1001-1250	46	25(54.3)		
Over 1250	40	26(65.0)		
**Duration of stay in Beijing(years)**				
Less 1	89	25(28.1)	4.075	0.130
1-5	123	51(41.5)		
Over 5	112	42(37.5)		
**Insurance coverage**				
Yes	20	14(70.0)	10.380	0.001
No	304	104(34.2)		

*Note: *309 (324 incidents) of the total respondents fell ill within the past two weeks(n = 324).

In order to further explore what socio-demographic factors play an important role in health seeking behavior among migrant workers, the multilevel logistic regression model was conducted by using "whether they had resorted to health service when they fell ill within the past two weeks" as a dependent variable, and education, work hours per day, monthly household income per capita, and insurance coverage as independent variables (Table 6). Health seeking behavior was measured as follows: one, if the migrant worker visited a doctor when he fell ill within the past two weeks, and zero, if he didn't go to visit a doctor. The multilevel model analysis revealed that health seeking behavior was significantly associated with insurance coverage. Meanwhile, monthly household income per capita and working hours per day also made a significant difference to the health seeking behavior among migrant workers in Beijing.

**Table 6:** Multilevel models on factors related to health seeking behavior among migrant workers who fell sick in the past two weeks

Variable	*β*	*SE*	*χ*^**2**^	*P*	OR
**Fixed part**					
Constant	-2.185	0.167	7.354	0.007	0.112
Education					
Primary school (illiteracy)	0.358	0.217	2.853	0.091	1.430
Secondary school (illiteracy)	0.420	0.109	2.523	0.112	1.522
High school or above (illiteracy)	0.453	0.165	3.301	0.074	1.573
Monthly household income per capita					
251-500(Less 250RMB)	0.448	0.167	4.458	0.035	1.565
501-750(Less 250 RMB)	0.577	0.156	8.201	0.004	1.781
751-1000(Less 250 RMB)	0.611	0.183	5.962	0.015	1.842
1001-1250(Less 250 RMB)	0.646	0.254	6.987	0.008	1.908
Working hours per day					
8-9(Less 8 hours)	0.145	0.102	1.377	0.241	1.156
10-11(Less 8 hours)	-0.008	0.003	3.951	0.047	0.992
12-13(Less 8 hours)	-0.013	0.009	4.252	0.039	0.987
Over13(Less 8 hours)	-0.532	1.163	9.214	0.002	0.587
Insurance coverage (Yes)	0.896	0.115	10.106	0.001	2.450
**Random part**					
Level 2 u_0jk_^2^	0.732	0.218	3.581	0.058	2.079
Level 1 scale parameter δ	1	0.000	-	-	-

*Note: *Category of each variable in the parentheses is the reference group. 309 (324 incidents) of the total respondents fell ill within the past two weeks(n = 324).

## Discussion

### Health issues of migrant workers in Beijing

In Beijing, the increasing migrant population has increased demand for the provision of various health services, including primary health care services, allied medical services, school health programs as well as overall health service manpower.

In this study, the two-week prevalence rate of the migrant workers was 13.1%, which was 4.6% lower than that of the total rural population (17.7%) reported by the national health services survey in 2008 [13], and also lower than that of migrant populations in other cities of China, such as Kunshan City, Jiangsu Province (18.2%) [14]. This phenomena is mainly due to most migrant workers are young and healthy compared to residents in receiving communities and sending communities. More serious conditions resulted in a migrant's return home to be looked after by family and to avoid the high medical and living costs in cities, which has the perverse effect of making the countryside export good health and re-import ill health [15].

A recent change is taking place among migrants in Beijing, which is the proportion of migrants move with their family members increases[3]. This study shows that more than half of the migrants bring their family to Beijing. With the household registration (*hukou*) system in China, rural-urban migrants are classified as temporary residents, irrespective of how long they stay in Beijing. They tend to rebuild 'a rural society' in the city, and establish 'a village-amidst-the-city'. With very few exceptions, migrants are frequently marginalized in urban communities and are targets of discrimination [16,17]. They usually live in poorly sanitized and overcrowded dormitories provided by their employers or in other shared accommodations [18,19]. In this survey, most of the migrant workers were of low socioeconomic status and they lived in rented housing on the city outskirts, while others lived in dormitory-style accommodations with public toilet facilities. Overcrowded, insalubrious living conditions may amplify the ease of spread of infectious diseases among the population. Poor living conditions and inattention to their own health make migrants vulnerable to long-term health problems [20].

### Factors associated with health seeking behavior and risk perception of migrant workers

Because their jobs were on short-term bases, migrant workers suffered from unstable lives, little social support and concern about their future [21]. When their expected residency in a given location is limited, the migrant workers are strongly discouraged to invest time and money in their temporary living places or employer-based insurance programs, or even to invest in their personal health and safety measures [22]. The completed study shows that less than 3% of migrants were covered by health insurance schemes, albeit even those would have very limited access to health services due to the low level of financial protection that these schemes provide [18]. This study shows that insurance coverage plays an important role in health seeking behavior, while 94.0% of the migrant workers don't have any insurance coverage in Beijing. This indicates that, to date, an overwhelming majority of this population has been uninsured and having to pay out-of-pocket health expenses. Moreover, the cost of medical care has risen dramatically in recent years, 20.3% of the migrants in this study stated that they would never seek any health care from medical institutions. A major driving force being that perverse incentives altered physicians' behavior toward self-interest at the expense of patients, even where professional ethics dictated otherwise [23]. The migrant workers are thus put in a disadvantaged position regarding access to health care services when working and living in the urban areas [24,25]. The means to provide effective health services for migrant populations has become an issue of urgency for the Chinese government and local city councils [26]. Expanding health coverage to migrants will be critical for effective prevention and control of epidemic diseases and for closing the widening gaps in health statuses across subpopulations in China. The success of health reforms will be shaped to a large extent by how migrant workers are incorporated into the rural or urban insurance schemes and how effective population health initiatives are in reaching the migrant population [27].

In this survey, the two-week visitation rate to doctors was 4.8%, only accounting for 36.4% of the migrant workers who fell ill within the past two weeks. This is 10.4% lower than that of rural populations, and 7.9% lower than that of urban populations reported by the national health services survey in 2008 [13]. Meanwhile, nearly one-fifth of the sick migrants who were recommended by physicians to have been hospitalized failed to receive medical treatment. According to self-reported reasons, the high cost of health service was a significant barrier to health care access. The multilevel model analysis also indicates that household income is a key factor in the utilization of health services. Being unable to pay, some people may choose to not seek health care services when they fall ill. Although migrant workers live temporarily in Beijing, they have to face the same problems as the farmers faced in rural China, which is medical expenditure has clearly become an important cause of transient poverty, and, indeed, one of the major poverty generators [28-30], Studies show that the high cost of health services and the lack of any health insurance have resulted in under-utilization of health care services among migrants, which had led to a series of ineffective health seeking behaviors such as unsupervised self-treatment, going to unregulated clinics, or 'just holding on' without seeking any medical care[8].

This survey indicates that the absence of health care awareness and risk perception among migrants deserves more attention. Nearly one-third of the migrant workers chose to take self-medication (33.3%) or no measures (30.3%) when they were ill within the two past weeks. Moreover, the Chi-square test result indicates that those with lower educational levels show a lower probability to utilize health services than that of the higher educated groups. Some of those thought their diseases weren't severe, and what is more noteworthy is that a small number of them thought they could treat the diseases themselves. During the interview, we found more than ten percent of the migrants (273 subjects) whose thought patterns usually reflected animistic and religious beliefs to some extent, and they had little knowledge or ability to prevent diseases and care for those in an unhealthy state, potentially resulting in grave health consequences in the long term.

Our investigation shows that working over time is common for migrant workers, nearly one-third of the total subjects work more than 12 hours per day, and most cannot rest on statutory holidays. Moreover, 31.2% work in the manufacturing and construction sectors, which belong to labor-intensive sectors, and high-risk jobs, work areas in which overtime will severely harm their physical and mental health [4,31]. The multilevel model analysis indicated that the longer working hours were associated with lower probability for health seeking behavior. This can also be seen in the self-reported main reasons for not seeking any medical care while they were ill as nearly one-third complained of having no free time. Our findings strongly suggest that attention should be paid to over-working in migrant populations. To maintain migrants well-being, the labor and social security sectors should design and implement appropriate regulations or laws to guarantee legal resting time for the migrant workers.

The different backgrounds and perspectives of local health care providers and patients require attention be paid to the potential difficulties of doctor-patient interaction. During the interview, we found that due to perceived discrimination and mistrust from medical professionals, some of the migrant workers were reluctant to resort to health services before they thought their diseases were serious enough to go to hospitals. Moreover, one-fifth of the migrant workers sought folk remedies from unlicensed private clinics. Because these practitioners often come from the same hometowns as migrant workers, familiarity was helpful in developing a good physician-patient relationship. Migrants tend to put their trust in the folk healers more than formally trained health care providers. However, most of the service providers in the so-called private clinics were unqualified practitioners in Beijing. They came from different rural areas of China and some of them used to be village doctors in their hometown. To avoid supervision from the local health administrative departments, they usually practiced secretly in their rental houses or provided home visiting services for migrant workers in Beijing. Moreover, through our field observations and investigation from the local health bureau, we found there existed a lot of disappointing facts regarding the so-called private clinics, including unsanitary conditions in medical facilities and a lack of modern medical equipment. Misdiagnosis and mistreatment may be inevitable and serious harm could be done to migrant workers when they seek health care services from these unlicensed clinics.

### Suggestions on solutions to the health seeking behavior dilemma of migrant workers

Adequate health care is one crucial consideration in a civil society, and every person should have the right to access care [32]. The migrants should not be deprived of entitlement to health benefits and community services because of their household registration status [8]. One of the main challenges to health care planners is to reach the most marginalized and vulnerable populations and to ensure universal access to affordable and equitable health care services [33]. While health insurance schemes will remain limited for the foreseeable future, attention should focus on providing affordable health care services to uninsured migrants [20]. Relevant policies of public medical insurance and assistance programs should be vigorously implemented for migrants. To further improve the health situation and risk perception among the migrant workers, and fundamentally solve their problem of health services access, proper attention and appropriate policies are especially recommended. Firstly, the central government should increase its investment in medical and health services for the migrant workers. The expenses for the provision of health care services for the migrant population should be incorporated into the state budget by means of government transfer payment. The responsibilities of all relevant governmental bodies, including public security, medical and health care institutions and the migrant population administration departments should be coordinated [34]. Secondly, to supply adequate basic medical and public health service for the migrants, the current capacity of service provision in community health service organizations needs to be expanded. The health care service institutions should be staffed in proportion to the number of both permanent resident and migrant population. The State should acknowledge the necessity for diversifying the health care workforce for the benefit of all populations. Thirdly, the government and concerned organizations should offer health education and improve social support for the migrants [35]. The health services providers in formal medical institutions should provide patient, considerate and professional services for migrants to help them increase their sense of attachment to their current communities. Professional help should be more readily accessible and affordable to the population so that demands of different levels can be satisfied [33,35].

## Conclusion

This survey contributes to our understanding of the health seeking behavior among Chinese rural-to-urban migrant workers and indicates that the state of the current health services system discourages migrant workers from seeking appropriate and quality care. A widespread challenge of China's public health system in terms of how to provide equitable access to the migrant workers emerges, and there is still a long way to go to ameliorate urban-rural inequality and integrate migrants into the urban society and social welfare network [36]. Feasible measures need to be taken immediately to reduce the risk of unhygienic practices, and equity should be assured in access to health care services among migrant groups.

The research is designed as descriptive rather than analytical, it offers a description of the migrant workers in Beijing, causal factors are undetermined through this cross-sectional survey, and over generalization is likely despite efforts to avoid it. A continuous survey is needed to update these data so as to generate more feedback for experience-based suggestions on improving the health seeking behavior of the migrant workers.

## Competing interests

The authors declare that they have no competing interests.

## Authors' contributions

WNL participated in the design of the study and drafted the manuscript. YCP participated in the design of the study and helped to edit, draft and revise the manuscript. WHC conceived the study and helped to draft the manuscript. HQZ assisted with editing and performed the statistical analysis. HPH participated in the statistical analysis and review of the manuscript. All authors read and approved the final manuscript.

## Pre-publication history

The pre-publication history for this paper can be accessed here:

http://www.biomedcentral.com/1472-6963/10/69/prepub
